# Fiber type-specific afferent nerve activity induced by transient contractions of rat bladder smooth muscle in pathological states

**DOI:** 10.1371/journal.pone.0189941

**Published:** 2017-12-21

**Authors:** Nahoko Kuga, Asao Tanioka, Koichiro Hagihara, Tomoyuki Kawai

**Affiliations:** Pharmacology Research Laboratory, Watarase Research Center, Kyorin Pharmaceutical Company, Limited, Nogi, Tochigi, Japan; Georgia State University, UNITED STATES

## Abstract

Bladder smooth muscle shows spontaneous phasic contractions, which undergo a variety of abnormal changes depending on pathological conditions. How abnormal contractions affect the activity of bladder afferent nerves remains to be fully tested. In this study, we examined the relationship between transient increases in bladder pressure, representing transient contraction of bladder smooth muscle, and spiking patterns of bladder afferent fibers of the L6 dorsal root, in rat pathological models. All recordings were performed at a bladder pressure of approximately 10 cmH_2_O by maintaining the degree of bladder filling. In the cyclophosphamide-induced model, both Aδ and C fibers showed increased sensitivity to transient bladder pressure increases. In the prostaglandin E2-induced model, Aδ fibers, but not C fibers, specifically showed overexcitation that was time-locked with transient bladder pressure increases. These fiber type-specific changes in nerve spike patterns may underlie the symptoms of urinary bladder diseases.

## Introduction

**Fig 1:**
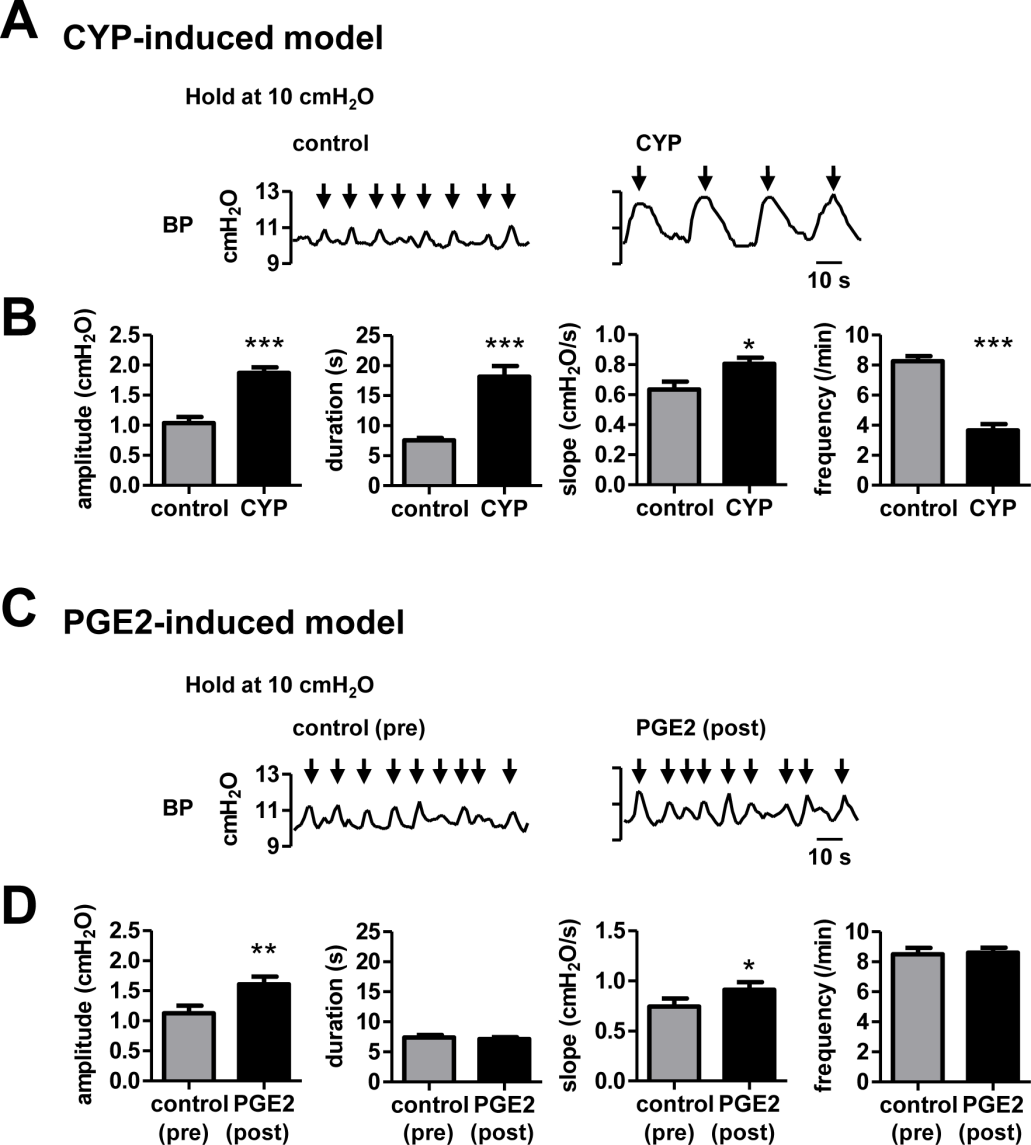
Comparison of transient BP increases between CYP-pretreated and PGE2-treated bladder. A, Representative traces of spontaneous transient BP increases (indicated by the arrows) at a holding BP of 10 cmH_2_O in normal (left) and CYP-pretreated bladder (right). B, Average amplitude, duration, slope, and frequency of transient BP increases. *P < 0.05, ***P < 0.001, Student’s *t* test (control, *N* = 20 rats; CYP, *N* = 10 rats). C, D, Similar to A and B but shows data before (control (pre)) and after application of PGE2 (PGE2 (post)). *P < 0.05, **P < 0.01, paired *t* test (*N* = 15 rats).

Bladder smooth muscle shows spontaneous transient (phasic) contractions. It has been shown that there are pronounced abnormal changes in the patterns of transient contractions in both human patients [1, 2] and animal disease models, including the cyclophosphamide (CYP)-induced cystitis [3, 4] animal model and animal models with detrusor overactivity induced by prostaglandin E2 (PGE2). Both the CYP-induced model and the PGE2-induced model have been shown to exhibit pathological increases in the amplitude of transient contractions of the bladder smooth muscle [3–6], as will be shown in Fig 1.

Bladder afferent nerves continuously sense the pathological phasic contractions of bladder smooth muscle and transmit the information regarding bladder conditions to the central nervous system. This has been considered as a major mechanism underlying pathological symptoms [7], such as urgency [8]. While early studies have reported the relationship between pathological contractions and bladder nerve activity [9–13], detailed characteristics of the physiological dynamics have not been fully elucidated.

Bladder afferent nerves are classified into two types, Aδ fibers and C fibers. Aδ fibers are generally thought to detect bladder filling under normal conditions, whereas C fibers are specifically activated under pathological conditions [14]. Consistently, several previous studies have shown that firing patterns of Aδ fibers, rather than C fibers, are time-locked with transient BP increases in normal animals [5, 15, 16]. We have previously shown that C fibers increase their activity levels in response to a gradual increase in bladder pressure (BP) in the presence of PGE2 in the bladder [5]. However, it remains unclear how fiber type-specific firing in response to transient BP increases is altered in models of pathological conditions.

To address this issue, we analyzed spike patterns of bladder afferent nerves in reference to the transient BP increases [17] observed in the rat CYP-induced cystitis model and the PGE2-induced model [5]. Compared with the PGE2-induced model, the CYP-induced model specifically includes painful behavior [18, 19] because of bladder inflammation [20, 21]. We demonstrate that different patterns of abnormal transient contractions in the two disease models lead to distinct firing patterns of afferent nerve fibers.

## Materials and methods

### Ethical approval

Experiments were performed with the approval of the Animal Experiment Ethics Committee of Kyorin Pharmaceutical Co. and were consistent with the Guide for the Care and Use of Laboratory Animals published by the US National Institutes of Health (NIH Publication, 8th Edition, 2011).

### Animals

A total of 31 adult female Sprague–Dawley rats (8–11 weeks old, weight 260–320 g; Charles River Laboratories Japan, Inc., Yokohama, Japan) were used in this study. The rats were housed in a transparent Plexiglas home cage (27 cm × 40 cm × 19 cm) with free access to water and food pellets under standard laboratory conditions at a temperature of 23°C with a 12 h light–12 h dark cycle. The estrous cycle of the female rats was not taken into consideration.

### Animal models

The CYP-induced model was generated by intraperitoneal administration of CYP (Shionogi Pharma, Osaka, Japan) dissolved in saline (100 mg kg^−1^, 10 ml kg^−1^), while control animals received an equal volume of saline, 24 h before the experiments commenced. The health of the animals was monitored at the next day of the administration. CYP is metabolized to acrolein, which is excreted in the urine and produces bladder inflammation [18]. The PGE2-induced model was produced by acute intravesical administration of PGE2 solution (100 μM; Sigma, St. Louis, MO).

### *In vivo* extracellular recordings

*In vivo* extracellular recordings were performed as described in our previous study [5]. The rats were anesthetized with urethane (1.5 g kg^−1^, s.c.; injection volume, 0.75 ml (100 g body weight^−1^); concentration, 20% w/v in saline; Wako, Osaka, Japan). Adequate depth of anesthesia was confirmed by the absence of pedal and corneal reflexes. The breathing rate and body temperature were continuously monitored throughout the recording. In this study, no humane endpoints were used during the anesthesia. The mortality rate during the 5-hours anesthesia was 0%. The rat was fixed on its back on a flat heat pad to maintain body temperature at 37.5°C. The trachea was cannulated to facilitate respiration. The pelvic structure was exposed by lower left abdominal incision and the pelvic nerve was dissected from the surrounding tissue proximal to the major pelvic ganglion. A bipolar silver electrode was placed on the pelvic nerve, and the pelvic structure around the electrode was sealed with Kwik-Cast™ (World Precision Instruments Ltd., Sarasota, FL). A double-lumen catheter was inserted into the bladder through the dome. The inner catheter was connected to a filling pump (Aladdin Pump; World Precision Instruments Ltd.) for infusion of saline or PGE2 solution. The outer catheter was connected to a pressure transducer (DX-100; Nihon Kohden, Tokyo, Japan) for measurement of BP. A lumbar laminectomy was performed and the dura was removed. The surface of the spinal cord was covered with paraffin oil. Bilateral L6 and S1 dorsal roots were cut close to their entrance to the spinal cord. The left L6 dorsal root was split into thin bundles and a fine filament was isolated from the bundle to obtain spike activity at the single-unit level. The filaments were teased until a maximum of three clearly different unitary action potentials were evoked by electrical stimulation (0.4 msec square wave pulses) of the pelvic nerve. The extracellular signals were recorded by placing the teased fiber on a bipolar silver electrode, and were pre-amplified (×10) and filtered (30–3000 Hz). The signals were then amplified (×10,000) and filtered using a 50 Hz noise eliminator (Hum Bug; Quest Scientific, North Vancouver, BC, Canada). BP and afferent activity were digitized at a sampling rate of 20 kHz, using a PowerLab data acquisition system (PowerLab 8/30; AD Instruments, Castle Hill, NSW, Australia).

### Experimental procedure

First, the spike activity in response to bladder distention was recorded from the fine filament for identification of an afferent nerve innervating the bladder. The bladder was filled with saline until the BP reached 30 cmH_2_O. In the CYP-induced animal model, the bladder was modestly filled until a BP up to 15 cmH_2_O was reached. Otherwise, the bladder response became severely unstable if the BP was increased to 30 cm H_2_O, due to bladder shrinkage caused by inflammation. The saline was then drained from the outer catheter. Next, the unit activity was recorded while the BP was held at 10 cmH_2_O. A T-shaped stopcock was placed between the outer catheter and the pressure transducer, and the other exit was connected with a tube to a platform 10 cm high. Saline was infused for 30 min at a rate of 2 ml/h. In the experiments for the PGE2-induced model, saline was replaced with PGE2 for 30 min. The last 5 min of recording of saline or PGE2 infusion was used for analysis. At the end of the experiment, the rats were euthanized with an overdose of sodium pentobarbital, and death was verified by monitoring cardiopulmonary arrest.

### Data analysis and statistics

We included some reanalyzed data from our published paper (*N* = 7 rats; Aδ fibers, *n* = 9; C fibers, *n* = 7) [5], which reduced the number of experimental animals required. To detect transient BP increases, the local maximum and minimum were detected throughout a BP trace. The difference in BP between a local maximum and the next local minimum was calculated as the amplitude of a transient BP increase. Amplitudes of less than 0.3 cmH_2_O were defined as noise signals and thus excluded from analyses. The duration of a transient BP increase was defined as the period between the times of two neighboring local minimum points. The multiunit data were analyzed based on the spike sorting algorithm, Wave_clus [22], and sorted into single unit data. The single units were classified as Aδ fibers and C fibers based on the half-maximal width of axonal spikes of individual units [5]. Units that were activated in response to bladder distention were included in the analyses. Instantaneous firing frequency was calculated with a bin size of 1 s. Average firing frequency at the time of the BP increase was computed by averaging all maximum firing rates detected within 3 s before and after the time, giving the local maximum of transient BP increases. Average firing frequency at the time of BP baseline was computed by averaging all firing rates detected within 1 s before and after the time, giving the local minimum BP.

**Fig 2:**
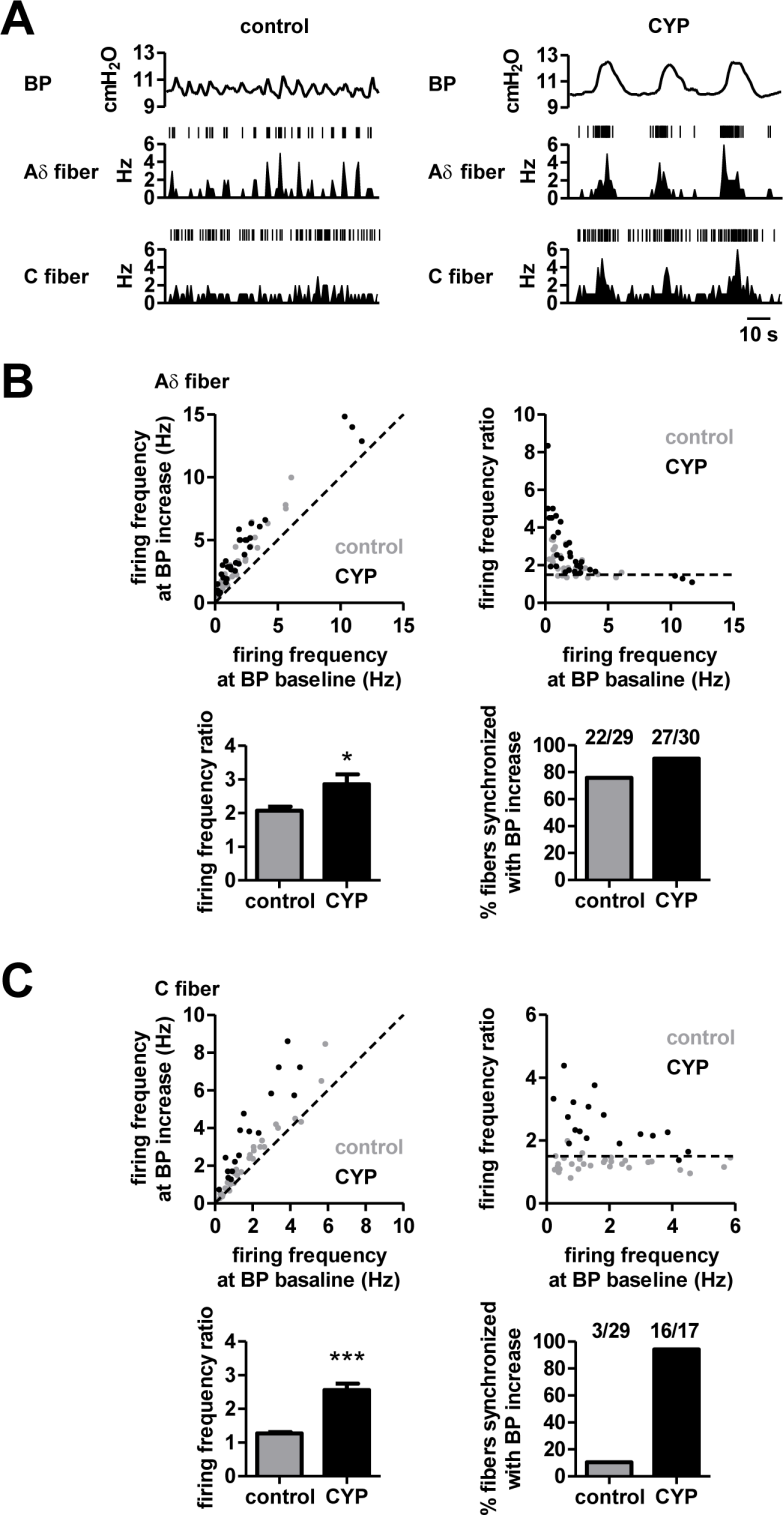
Firing patterns of Aδ and C fibers in response to transient BP increases in control and CYP-pretreated animals. A, (Top) Representative traces of spontaneous transient BP increases at a holding BP of approximately 10 cmH_2_O in control (left) and CYP-pretreated bladder (right). (Middle and bottom) The corresponding firing frequencies of representative Aδ fibers (middle) and C fibers (bottom) in control and CYP-pretreated animals. B, **(**Top left) In individual Aδ fibers, average firing frequencies were calculated at the time of transient BP increase (Y-axis) and at BP baseline (X-axis). Each dot represents one fiber (control, gray; CYP, black). The dotted diagonal line has a slope of 1, showing that both frequencies are similar. Bottom left, the ratio of average firing frequencies at BP increases to those at BP baseline. *P < 0.05, Mann–Whitney U test (control, *n* = 29 fibers from 17 rats; CYP, *n* = 30 fibers from 9 rats). (Top right) The ratio of each Aδ fiber was plotted as a function of its frequency at BP baseline. A synchronized fiber was defined as a fiber with a ratio > 1.5, which is represented by the dotted horizontal line. (Bottom right) The fraction of Aδ fibers classified as synchronized fibers. C, The same as B, but for C fibers. ***P < 0.001 (control, *n* = 29 fibers from 15 rats; CYP, *n* = 17 fibers from 8 rats).

In each fiber, the ratio of the firing frequency at BP increase to firing frequency at BP baseline was computed as a firing frequency ratio. A “synchronized fiber” was defined as a fiber in which the ratio exceeded 1.5 (Fig 2B and 2C, top right, the dotted horizontal line). This detection criterion was set based on our previous data that the ratio of firing frequency at the 1.96 standard deviations above the mean of firing frequency at BP baseline, which corresponds with a significance level of 5%, was 1.4 ± 0.052 (*n* = 23 fibers) [5].

The statistical significance of the results was evaluated using the Student’s *t* test, the paired *t* test, or the Mann–Whitney U test, as specified in the figure legends. Differences were considered significant at *P* < 0.05. All data are expressed as mean ± SEM.

## Results

### Transient BP increases differ between the CYP-induced and PGE2-induced animal models

Even when no external stimuli were applied to the bladder, BP showed ongoing transient increases with an amplitude of ~2 cmH_2_O, while BP was experimentally adjusted to approximately 10 cmH_2_O by infusion of saline into the bladder (Fig 1A, left, control). We first monitored how the transient BP increases were altered in the two animal models: (i) the CYP-induced model and (ii) the PGE2-induced model. In the CYP-induced model animals, there were significant increases in the amplitude, duration, and slope [23] of transient BP increases compared with control animals (control, *N* = 20 rats; CYP, *N* = 10 rats. Amplitude: control, 1.0 ± 0.10 cmH_2_O; CYP, 1.9 ± 0.088 cmH_2_O, Student’s *t* test, *P* < 0.001; Duration: control, 7.6 ± 0.36 s; CYP, 19.0 ± 1.7 s, Student’s *t* test, *P* < 0.001; Slope: control, 0.62 ± 0.056 cmH_2_O/s; CYP, 0.81 ± 0.038 cmH_2_O/s, Student’s *t* test, *P* < 0.05), whereas the frequency was reduced (control, 8.3 ± 0.34/min; CYP, 3.5 ± 0.40/min, Student’s *t* test, *P* < 0.001) (Fig 1B). To generate the PGE2-induced model, PGE2 was infused into the bladder during recording. The periods before and after PGE2 application were defined as control (pre) and PGE2-induced (post) periods, respectively. In PGE2-treated animals, the amplitude and slope of transient BP increases were increased compared with that before PGE2 application (*N* = 15 rats; Amplitude: control, 1.1 ± 0.12 cmH_2_O; PGE2, 1.6 ± 0.12 cmH_2_O, paired *t* test, *P* < 0.01; Slope: control, 0.74 ± 0.078 cmH_2_O/s; PGE2, 0.91 ± 0.074 cmH_2_O/s, paired *t* test, *P* < 0.05), while the duration and frequency remained unchanged (Duration: control, 7.4 ± 0.44 s; PGE2, 7.1 ± 0.29 s, Frequency: control, 8.5 ± 0.42/min; PGE2, 8.6 ± 0.34/min, paired *t* test) (Fig 1C and 1D).

### Synchronized activity of afferent fibers innervating the bladder in response to transient BP increases

We first confirmed that bladder afferent nerves under normal conditions showed spiking activity induced by transient BP increases. Extracellular recordings were performed from afferent nerves of the L6 dorsal roots concurrently with BP recording (Fig 2A, left, control). Bladder afferent nerves were classified into Aδ fibers and C fibers based on the half-maximal width of axonal spikes of individual units, as described in our previous study [5]. In each fiber, averaged firing frequencies at the time of transient BP increases and at the BP baseline were measured (Fig 2B and 2C, top left, gray). We defined a fiber as “synchronized fiber” when the firing frequency ratio, which represents quantifies the degree of activity change against transient BP increases and represents how sensitively each fiber unit emits spikes time-locked to the transient BP increases, exceeded 1.5 (Fig 2B and 2C, top right, the dotted horizontal line; for more detail, see Methods). Under control conditions, 75.9% and 10.3% of Aδ fibers and C fibers respectively, were classified as synchronized fibers (Fig 2B and 2C, bottom), suggesting that Aδ fibers are more sensitive to transient BP increases than C fibers under normal conditions.

### Synchronized fiber populations associated with transient BP increases differ between the CYP-induced and the PGE2-induced model

**Fig 3:**
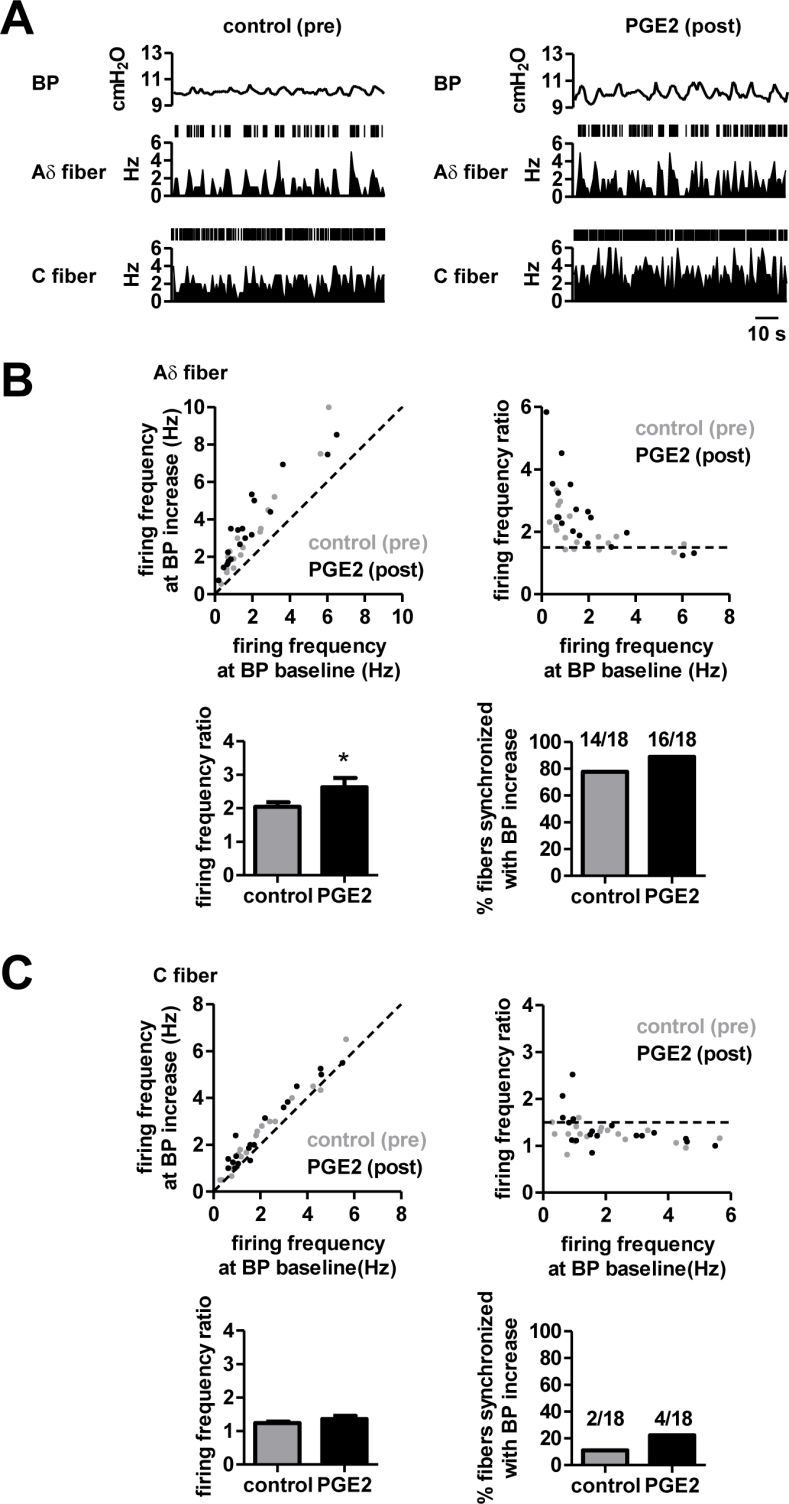
Firing patterns of Aδ and C fibers in response to transient BP increases in animals with PGE2 infusion into the bladder. All graphs are as in Fig 2 but data obtained before and after PGE2 application are shown as control (pre) and PGE2-induced (post) activity, respectively. *P < 0.05, paired *t* test (Aδ fibers, *n* = 18 fibers from 12 rats; C fibers, *n* = 18 fibers from 10 rats).

Having established the firing properties of the two fiber types associated with transient BP increases, we next examined whether the BP increases and firing patterns were altered in pathological states. In the CYP-induced animal model, both Aδ fibers and C fibers exhibited increased spiking activity time-locked with such transient BP increases as shown in Fig 2A, right panel. For Aδ fibers, the majority (75.9%) of fibers were synchronized to transient BP increases under control conditions and the CYP-induced model showed an increase in this proportion to 90.0% (Fig 2B, bottom right), with a significant increase in the average frequency ratio (Fig 2B, bottom left). For C fibers, the majority (94.1%) of fibers also became synchronized fibers, with a significant increase in the average frequency ratio (Fig 2C). Taken together, the CYP-induced model exhibited enhanced synchronized activity to transient BP increases in both Aδ fibers and C fibers. Notably, the degree of activity change against transient BP increases was more pronounced in C fibers than in Aδ fibers. Using the same recording configuration, we examined afferent nerve activity in the PGE2-induced animal model. For Aδ fibers, the proportion of synchronized fibers was increased from 77.8% to 88.9% by application of PGE2, with a significant increase in the average frequency ratio (Fig 3B). In C fibers however, no marked increase in the proportion of synchronized fibers was detected in the presence of PGE2 and the average frequency ratio remained unchanged (Fig 3C). Taken together, these results suggest that Aδ fibers are able to sense transient BP increases in PGE2-induced overactive bladders, whereas C fibers are insensitive.

### Firing patterns of Aδ fibers and C fibers show distinct temporal relationships to pathological BP increases

**Fig 4:**
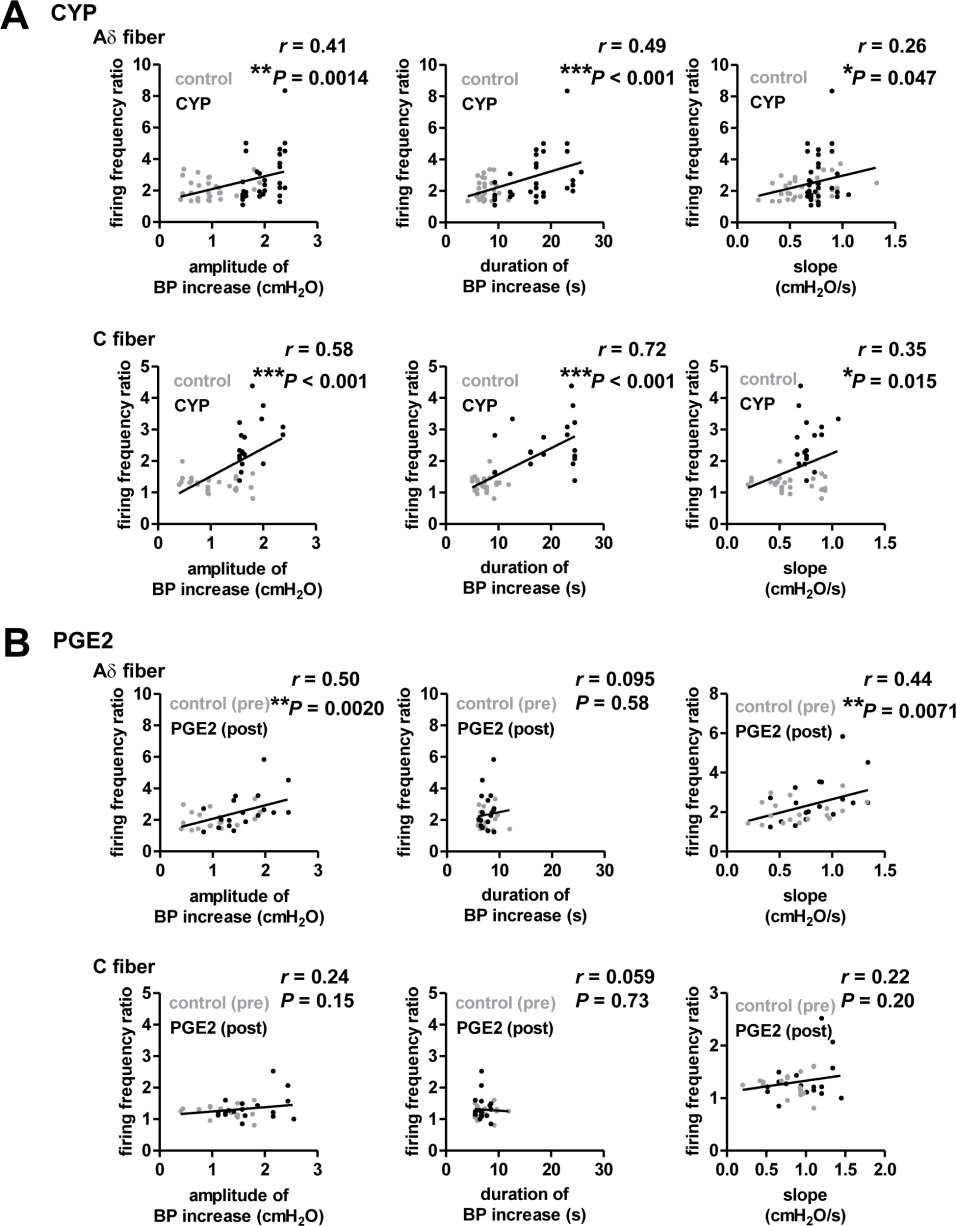
Relationship between transient BP increases and afferent nerve activity. A, The frequency ratio of each Aδ fiber (top) and C fiber (bottom) was plotted against the average amplitude (left), duration (middle), and slope (right) of transient BP increases in control (gray) and CYP-pretreated (black) animals. The solid line represents the linear regression line. B, The same as A, but for control (gray) and PGE2-treated (black) animals.

To further analyze how spiking activity of these fibers is related to detailed changes in transient BP increases, the frequency ratio of each type of fiber was plotted against the corresponding amplitude, duration, and slope of transient BP increases. In the CYP-induced model, which showed prominent increases in the amplitude and duration of transient BP increases (Fig 1A and 1B), the frequency ratios were significantly correlated with amplitude, duration, and slope in both Aδ fibers (amplitude, *r* = 0.41, *P* = 0.0014; duration, *r* = 0.49, *P* < 0.001; slope, *r* = 0.26, *P* = 0.047, Fig 4A) and C fibers (amplitude, *r* = 0.58, *P* < 0.001; duration, *r* = 0.72, *P* < 0.001; slope, *r* = 0.35, *P* = 0.015, Fig 4A). In the PGE2-induced model, in which the amplitude was specifically increased without changing the duration (Fig 1C and 1D), the frequency ratio of Aδ fibers before and after PGE2 application correlated with the increased amplitude and slope, but not the duration (amplitude, *r* = 0.50, *P* = 0.0020; duration, *r* = 0.095, *P* = 0.58; slope, *r* = 0.44, *P* = 0.0071, Fig 4B). On the other hand, such transient BP increases in the PGE2-induced model were not sufficient to trigger activation of C fibers.

**Fig 5:**
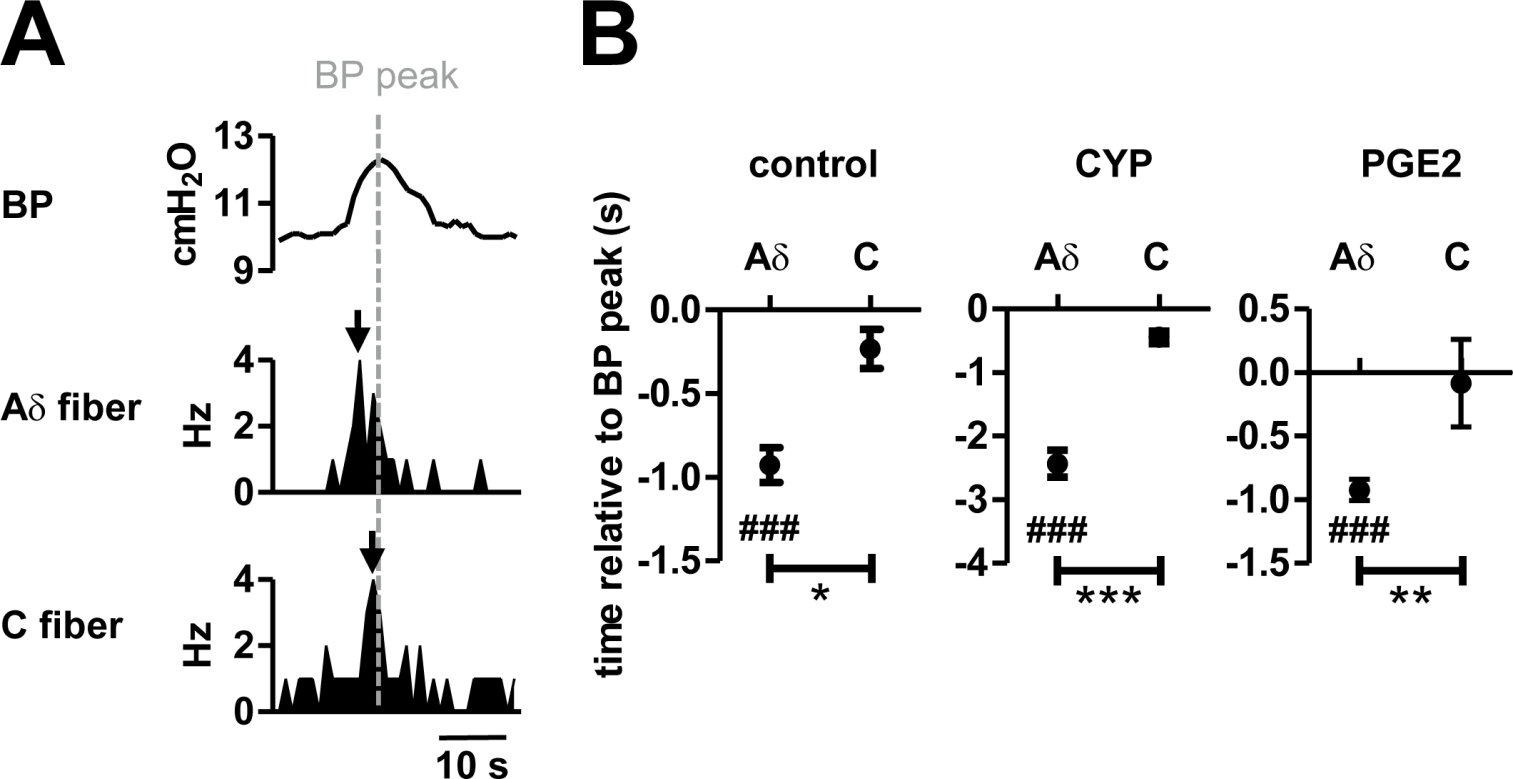
Temporal relationship between transient BP increases and afferent nerve activity. A, Spikes were aligned to the peak of each cycle of transient BP increases and average firing rates relative to the BP peak were calculated. An averaged BP trace aligned to the BP peak and the corresponding changes in average firing rates of an Aδ fiber and a C fiber are shown. The time at the maximum firing rate was defined as latency, shown as black arrows. The dotted vertical line represents the time of the BP peak. B, The average latency relative to the BP peak in two fiber types in control, CYP-pretreated, and PGE2-treated animals; # represents a significant difference from 0 within the group, defined by the paired *t* test, ###*P* < 0.001; * represents a significant difference between two groups defined by the Student’s *t* test, *P < 0.05, **P < 0.01, ***P < 0.001 (control Aδ fibers, *n* = 22 fibers from 15 rats; control C fibers, *n* = 3 fibers from 3 rats; CYP Aδ fibers, *n* = 27 fibers from 9 rats; CYP C fibers *n* = 16 fibers from 8 rats; PGE2 Aδ fibers, *n* = 16 fibers from 10 rats; PGE2 C fibers, *n* = 4 fibers from 3 rats).

We also analyzed the temporal relationships between transient BP increases and firing patterns of afferent fibers. The relative time intervals between the BP peak and the BP peak-triggered maximum firing rate of individual fibers were detected and defined as latency (Fig 5A). The average latency of Aδ fibers was negative and significantly less than zero under control conditions (*P* < 0.001), meaning that firing rates of Aδ fibers reached their peak values prior to the peak of corresponding transient BP increases (Fig 5B). On the other hand, the latency of C fibers was not different from zero under control conditions (*P* = 0.18). Taken together, these observations indicated that Aδ fibers begin firing at each cycle of transient BP increase earlier than C fibers. This temporal relationship is preserved even in pathological states (Aδ fibers: CYP, *P* < 0.001; PGE2, *P* < 0.001. C fibers: CYP, *P* = 0.069; PGE2, *P* = 0.82). The latency of Aδ fibers in the CYP-induced model (−2.4 ± 0.21 s) was significantly less than those of the controls and the PGE2-induced model animals. This is likely due to the fact that the duration of transient BP increases is much longer in the CYP-induced model (Fig 1).

## Discussion

In this study, we tested fiber type-specific changes in spike patterns in response to transient BP increases in the two animal models. Previous studies have shown that intravesical infusion of 100 μM PGE2 induced increased urinary frequency, and decreased micturition interval and bladder volume [6, 24], increased micturition BP and basal BP [24] (the dosage at tens of micromolar could induce same effects [25, 26]) in awake rats, and increased activity of C fibers [5, 27] and Aδ fibers with high threshold mechanosensitivity [5] in urethane-anesthetized rats. In humans, intravesical administration of PGE2 into the bladder caused decreased bladder capacity and increased micturition pressure, leading to bladder instability, urgency, urinary frequency [28]. In the 100 mg/kg CYP-induced model, a dosage used in this study, conscious rats show painful behavior [18, 19], increased urinary frequency, and decreased bladder volume [29]. In addition to these previous findings, this study demonstrated that the CYP-induced cystitis model showed increased synchronicity with transient BP increases in both Aδ fibers and C fibers. In particular, the recruitment of C fibers was more prominent compared with the PGE2-induced animal model.

As shown in Fig 1, we found that transient BP increases observed at a baseline BP of 10 cmH_2_O differed between the two animal models. The CYP-induced model showed a reduction in the total frequency with significant increases in the amplitude and duration of individual BP increases, whereas the PGE2-induced model showed an increased amplitude while the entire frequency remained unchanged. It has been reported that the CYP-induced model exhibits a marked increase in inflammatory factors [20, 21] in the bladder and disruption of the urothelium barrier [20], causing sensitization to irritants in the urine, whereas the PGE2-induced model has no disruption of the urothelium barrier. Although precise action sites of intravesical PGE2 remains unclear, both direct effect on bladder smooth muscle and indirect effects on the urothelium and/or suburothelium are considered to be involved in the PGE2-induced transient BP increases. A direct effect is supported by the evidence that application of PGE2 induces intracellular Ca^2+^ increase in bladder smooth muscle cells and facilitates spontaneous contraction of bladder strips without the urothelium and suburothelium [30]. Indirect effects are mediated by Acetylcholine and ATP released from the (sub)urothelium [31–34]. This ATP release can be induced by PGE2 through the release of neurotransmitters such as tachykinins from the afferent nerves [25, 35, 36], which can also evoke the contraction of the bladder smooth muscle [37, 38]. Such differences in biochemical and anatomical mechanisms between the two animal models are likely to account for the distinct patterns of the transient BP increases observed here.

The difference in the sensitivity to transient BP increases between Aδ fibers and C fibers may be explained by their intrinsic firing properties. Distinct firing modes of afferent Aδ fibers and C fibers have been reported, including: (1) tonic firing, i.e., sustained spikes during external stimuli, and (2) phasic firing, i.e., transient spikes in response to the onset of external stimuli [7]. Under control conditions, Aδ fibers showed tonic firing patterns time-locked with transient BP increases, which was further enhanced in CYP-induced animals (Fig 2B). On the other hand, C fibers have been reported to show phasic firing [7], which may account for their insensitivity against transient BP increases under control conditions (Fig 2C). Our data indicated that C fibers could also switch their firing patterns to a tonic-like firing mode in the CYP model, consistent with a previous report [39]. These different firing modes may be supported by distinct expression patterns and/or activation levels of certain types of ion channels [7].

Other possible mechanisms can be considered based on the results of the correlation data shown in Fig 4. In the PGE2-induced model, Aδ fibers, but not C fibers, could sense amplification of the transient BP increase cycles, with no change in duration (Fig 4B). In the CYP-induced model, which exhibited pronounced increases in both amplitude and duration of transient BP increases, activity levels of C fibers, as well as Aδ fibers, were correlated with the magnitude of the transient BP increases (Fig 4A). Furthermore, the results shown in Fig 5 indicate that firing of Aδ fibers can precede that of C fibers in each cycle of transient BP increases. Taken together with the fiber type-dependent tonic firing described above, our data consistently demonstrated that Aδ fibers are more sensitive to subtle changes in BP increases. These physiological characteristics are likely due to their distinct expression patterns of mechanosensitive channels, such as transient receptor potential (TRP) channels [40], and/or their anatomical projection patterns. In particular, Aδ fibers project to the smooth muscle layer and C fibers project throughout all layers of the bladder, including the urothelium, the suburothelial space, and the smooth muscle layer [41]. The preferential innervation of Aδ fibers onto the smooth muscle layer may be a mechanism by which Aδ fibers can detect smaller and more rapid changes in BP increases than C fibers.

Based on our observations, distinct physiological roles for these two fiber types and the consequences of abnormal changes should be considered. It is generally assumed that Aδ fibers sense bladder filling and are primarily activated to maintain normal bladder function, whereas C fibers show elevation of excitation levels specifically under pathological conditions associated with urinary urgency, frequency, and bladder pain [14]. Our previous study showed that Aδ fibers, rather than C fibers, are more sensitive to BP increases induced under normal conditions with a BP of ~10 cmH_2_O [5]. The present study showed that C fibers, in addition to Aδ fibers, increased their excitation levels by synchronizing with ongoing transient BP increases in the CYP-induced model. These detailed physiological analyses confirm that non voiding contractions, represented by transient BP increases, are encoded by a combination of unique characteristics of firing pattern bladder afferent nerve populations in pathological states.

## Conclusions

We revealed fiber type-specific activation of bladder afferent nerves associated with transient BP increases in the CYP-induced and PGE-induced models. In response to a growing demand for better treatment of urological diseases, our observations may help development of novel therapeutics that could ameliorate discomfort, by targeting of neurophysiological substrates.
